# Dihydropyridine Calcium Channel Blockers Suppress the Transcription of PD-L1 by Inhibiting the Activation of STAT1

**DOI:** 10.3389/fphar.2020.539261

**Published:** 2021-01-13

**Authors:** Xiaohui Pan, Run Li, Hongjie Guo, Wenxin Zhang, Xiaqing Xu, Xi Chen, Ling Ding

**Affiliations:** ^1^ Zhejiang Province Key Laboratory of Anti-Cancer Drug Research, Institute of Pharmacology and Toxicology, College of Pharmaceutical Sciences, Zhejiang University, Hangzhou, China; ^2^ The Second Clinical College, Zhejiang Chinese Medical University, Hangzhou, China

**Keywords:** dihydropyridine calcium channel blockers, NSCLC, STAT1, PD-L1, transcription

## Abstract

Programmed death ligand 1 (PD-L1) which is upregulated in various epithelial tumors, plays a central role in the evasion of the immune system. In addition to monoclonal antibodies that blocking PD1/PD-L1 axis, finding small molecule compounds that can suppress PD-L1 expression might be another substitutable strategy for PD1/PD-L1 based therapy. Here, we found that dihydropyridine calcium channel blockers dose-dependently reduced the expression of PD-L1, both in the cytoplasm and cell surface. IFNγ induced PD-L1 transcription was consistently suppressed by Lercanidipine in 24 h, whereas, the half-life of PD-L1 protein was not significantly affected. IFNγ trigged significant STAT1 phosphorylation, which was eliminated by Lercanidipine. Similarly, STAT1 phosphorylation could also be abolished by extracellular calcium chelating agent EGTA and intracellular calcium chelator BAPTA-AM. Furthermore, Lercanidipine enhanced killing ability of T cells by down-regulating PD-L1. Taken together, our studies suggest that calcium signal is a crucial factor that mediates the transcription of PD-L1 and regulation of calcium can be used as a potential strategy for PD-L1 inhibition.

## Introduction

The programmed death protein 1 (PD-1) and its ligand (PD-L1) are negative inhibitory signaling molecules, which play a key role in tumor immune escape. PD-L1 binds to either PD-1 or CD80 receptors on T cells, B cells, dendritic cells, and natural killer cells to inhibit their proliferation, cytokine release, and cytolytic activity. Blocking PD-1/PD-L1 coinhibitory ligation with monoclonal antibodies has achieved impressive clinical results in the treatment of several types of tumors (Buchbinder and Hodi, 2016; Ding et al., 2019). To date, six kinds of monoclonal antibodies of PD-1/PD-L1 have been approved by FDA, for the treatment of non-small cell lung cancer (NSCLC), melanoma, Hodgkin’s lymphoma and gastric cancer and more. However, the arguments to search for alternatives to mAbs in immunoncology exist. Antibodies are not orally bioavailable and their high molecular weight leads to poor diffusion, especially in large tumors. The Fc portion of IgG antibodies can interact with various receptors on the surface of different cell types, which affects their retention in the circulation. Further, mAbs are immunogenic and can lead to irAEs with deadly outcomes, albeit in rare cases (Konstantinidou et al., 2018). Given that PD-L1 is frequently over-expressed in various cancers (Dong et al., 2002; Audrito et al., 2017; Bertucci and Goncalves, 2017), finding small molecular compounds that suppress PD-L1 expression might be an efficient strategy. Recent studies have found that eIF4F (Cerezo et al., 2018), BRD4 (Zhu et al., 2016) are respectively associated with the translational and transcriptional regulation of PD-L1, furthermore, their respective inhibitors silvestrol, JQ1 all show obvious tumor suppressive effects, which suggests that there is large development space to excavate.

It has been widely recognized that calcium signal is a crucial regulator of processes related to tumor progression (Clapham, 2007; Roderick and Cook, 2008). Numerous studies have shown that the progression of tumors is often accompanied by the changes of calcium or driven by calcium, and specific calcium signaling pathways encourages the establishment of the tumor microenvironment (Monteith et al., 2017). At the same time, the expression of specific calcium channels and pumps is often observed in tumors (Monteith et al., 2007). Although there have been many studies on the role of calcium in tumors, there are still few studies on the regulation of calcium on tumor immunity. For example, its studies on PD-L1 have not been reported.

In our study, we found that dihydropyridine calcium channel blockers can inhibit the transcription levels of PD-L1 by inhibiting the phosphorylation of STAT1 which is widely regarded as the transcription factor of PD-L1 (Zerdes et al., 2018). As one of L-type calcium channel (LTCC) blockers, Lercanidipine inhibits calcium influx and downstream calcium signaling, and in this way Lercanidipine exhibits its biological function. Through verification, the decrease of cytosolic calcium or the inhibition of its downstream protein kinase CAMKII indeed inhibit the level of PD-L1 and enhance killing ability of T cells on tumor cells. Our results not only show that calcium signaling involves the gene expression, but also improve the understanding of the mechanism of regulation of PD-L1.

## Material and Methods

### Antibodies and Reagents

FITC-conjugated CD274 mouse monoclonal antibody (#558065), FITC-conjugated CD47 (#556045) and FITC mouse IgG were from BD Biosciences. FITC anti-human MHC Ⅰ (#343303), FITC anti-human CD8a (#300906) and FITC mouse IgG1 *κ* isotype control (#400110) were from Biolegend. Anti-PD-L1 (#13684), anti-Phospho-STAT1 (Tyr701) (#7649), anti-Phospho-STAT1 (Ser727) (#8826) and anti-CAMKII (#3362) antibodies were from Cell Signaling Technology. Anti- STAT1 p84/p91 (sc-346), anti-AKT1/2/3 (sc-8312), anti-P-AKT1/2/3 (Thr308) (sc-16646), anti-ERK (sc-94) and anti-P-ERK (sc-101761) antibodies were from Santa Cruz Biotechnology. Anti-GAPDH (db106) was from diagbio. Lercanidipine (PubChem CID: 65866; purity > 99%), Amlodipine (PubChem CID: 60496; purity > 99%), Azelnidipine (PubChem CID: 65948; purity > 99%), Verapamil (PubChem CID: 2520; purity > 99%), Diltiazem (PubChem CID: 39186; purity > 99%), BAPTA-AM (PubChem CID: 2293; purity > 99%), AMG-517 (PubChem CID: 16007367; purity > 98%), KN-93 (PubChem CID: 5312122; purity > 99%), MG132(PubChem CID: 462382; purity > 97%) and Chloroquine (PubChem CID: 6301; purity > 99%) were from TargetMol. Sulforhodamine B (#230162) was from Sigma-Aldrich. Fluo-4 AM (S1060) was from Beyotime.

### Cell Culture

All the cell lines were purchased from Cell Bank of Shanghai Institutes for Biological Sciences, Chinese Academy of Sciences (Shanghai, China). The NCI-H1299 and NCI-H460 cell lines (NSCLC) were maintained in RPMI 1640 (Gbico) medium with 10% fetal bovine serum (FBS; Gbico). All cells were maintained at 37°C in a 5% CO_2_ incubator.

### Western Blot

Protein samples were separated by SDS-PAGE, and then they were transferred to PVDF membranes. Membranes were blocked in 5% milk and TBST (150 mM NaCl, 10 mM Tris-HCl at pH 7.6, and 0.1% Tween 20) for 1 h, and incubated with primary antibodies overnight at 4°C. Washed three times with TBST for 25 min, membranes were incubated with secondary antibody (1:5,000) at room temperature for 1 h, and then washed three times in TBST again. The protein bands were analyzed by chemiluminescence using ECL detection reagent.

### Cell Survival Assay

Cells were plated at a density of 3 × 10^3^ cells per well in 96-well plates and allowed to adhere for 24 h then cells were exposed to special concentrations of inhibitors (10 μM) for 24 h. Cells were harvested and fixed by 10% TCA for 1 h or overnight at 4°C. After removing the media, cells were washed five times by PBS, and they were subsequently stained by sulforhodamine B (SRB). Following dye incorporation, fluorescence was measured at 499 nm with the SpectraMax M5 (Molecular Devices). The situation of cell proliferation for each well was calculated.

### SiRNA-Mediated Silencing

NCI-H1299 cells were plated at 50% confluency in 6-well plates for 24 h, then cells were transfected with transfection reagent JetPRIME (Polyplus, #114-15), Jet PRIME Buffer (Polyplus, #712-60) and STAT1 (CaMKII) siRNA or siRNA-negative control (Jet PRIME Buffer (Polyplus, #712-60); 200 µL, JetPRIME 2 μL, 20 μM siRNA: 2.5 μL for per well) for 24 h. The siRNA sequences used in the study are provided in Supplementary Table S1.

### RNA Isolation and Quantitative Real-Time PCR

Total RNA was extracted using TRIzol reagent (Invitrogen, #15596026), and it was further purified according to standard protocols. Single-strand cDNA was synthetized by using TransScript One-Step gDNA Removal and cDNA Synthesis SuperMix (TRAN, #AT311-03). Quantitative RT-PCR was accomplished with SYBR-Green kit (Bio-Rad, #172-5124), and its’ accuracy can be judged by melting curves and Repeated sample. Beta-actin was used as the normalizing gene, and calculation of the data all needs to normalize to its’ mRNA levels. The primers used are provided in Supplementary Table S2.

### Flow Cytometry

NCI-H1299 cells were collected, then they were washed twice with cold PBS and stained with FITC-conjugated CD274 (CD47, MHC Ⅰ) in 0.2% BSA at 4°C for 2 h (5 μL/2 × 10^5^ cells in 100 μL 0.2% BSA). Next, they were washed with PBS again, filtered through membrane before detecting by BD FACSuite TM (BD bioscience). The data were analyzed by one-way ANOVA with Dunnett’s post hoc test.

### Ca^2+^ Measurements

For intracellular Ca^2+^ measurements using Fluo-4 AM, NCI-H460 cells were cultured in 6-well plates and allowed to adhere for 24 h. Then cells were exposed to special concentrations of inhibitors and IFNγ for 24 h. NCI-H460 cells were collected, then they were washed three times with cold PBS and incubated with 2 μM Fluo-4 AM for 1 h at 37°C. Next, they were washed with PBS again and detected by BD FACSuite TM (BD bioscience).

### T Cell-Mediated Cancer Cells Killing Assay

The fresh heparinized blood was diluted by using ice cold PBS. Then the diluted blood was slowly added to the surface of the Ficoll-Paque solution (17-1440-02; GE). By density-gradient centrifugation with Ficoll, peripheral blood mononuclear cells (PBMC) were separated. Next, PBMC was washed by PBS with 0.1% BSA and 2 mM EDTA. Finally, PBMC, resuspend in TexMACS™ GMP Medium (170-076-309; Miltenyi Biotec), was stimulated about 2–3 days with ImmunoCult Human CD3/CD28/CD2 T cell activator (10970; STEMCELL Technologies) and IL-2 (30 U/mL; PeproTech) to be activated T cells.

The cancer cells were seeded in 96-well plate and permitted to grow for 24 h, then they were treated with IFNγ, other pharmacological inhibitors for 24 h. Next, the drugs was removed and activated T cells was added to cancer cells for 24–48 h, and the ratio of cancer cells to T cells was around 1 to 5 or 10. Finally, T cells and cell fragments were eliminated carefully by PBS, and the living cancer cells were stained with Crystal Violet or SRB so that their survival condition can be observed.

### Statistical Analysis

All of the data are presented as mean ± SEM. Student *t*-test was used to determine Statistical differences (two groups). The data were analyzed by one-way ANOVA with Dunnett’s post hoc test (more than two groups) with PRISM (GraphPad 7.00 Software). *p* values below 0.05 were considered as significant. All figures were acquired by using GraphPad Prism software (GraphPad Software). The images were quantified with Image-Pro Plus 6.0 software.

## Results

### Dihydropyridine Calcium Channel Inhibitors Suppress the Expression of PD-L1 in Lung Cancer

**FIGURE 1:**
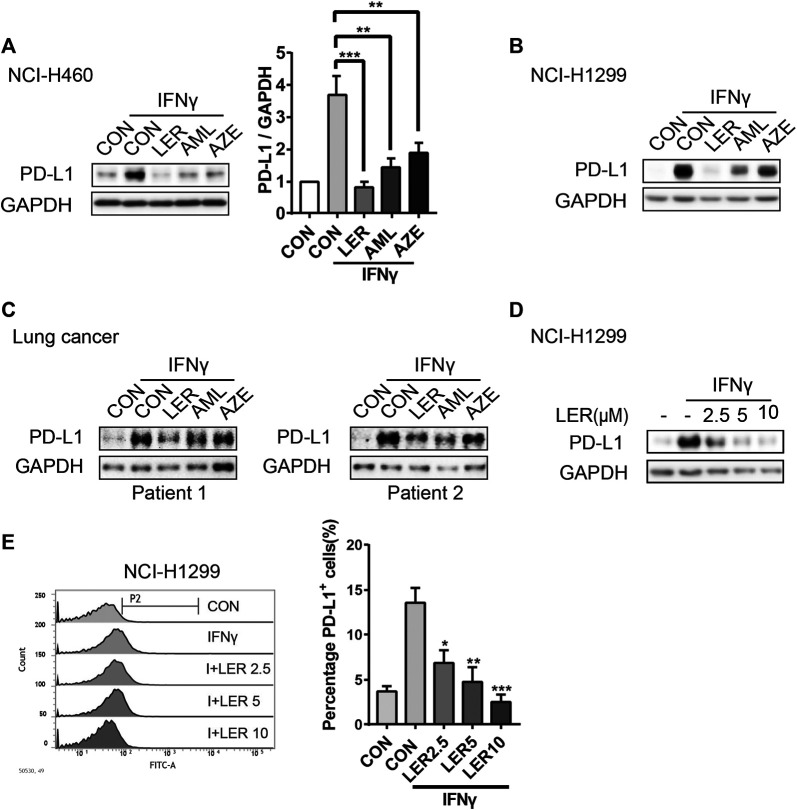
Dihydropyridine calcium channel blockers inhibited IFNγ induced expression of PD-L1. **(A–C)** The expression of PD-L1 protein was measured by Western blot in NCI-H1299, NCI-H460 cells and primary lung cancer when treated with three kinds of dihydropyridine calcium channel blockers (10 μM) and IFNγ (10 ng/ml) for 24 h. The relative protein level of PD-L1 in NCI-H460 was analyzed quantitatively on the right. **(D)** NCI-H1299 cells were treated with and Lercanidipine (2.5, 5, and 10 μM) for 24 h, and the expression of PD-L1 was analyzed by Western blot. n = 3. **(E)** Surface PD-L1 expression on NCI-H1299 treated with both IFNγ (10 ng/ml) and the indicated doses of Lercanidipine (2.5, 5, and 10 μM) was determined by flow cytometry. Cells were estimated for PD-L1 or mouse IgG control antibody. Data were the mean ± SEM of triplicate experiments. The data were analyzed by one-way ANOVA with Dunnett’s post hoc test. ***, *p* < 0.001; **, *p* < 0.01; *, *p* < 0.05.

When tumor cells are infiltrated by cytotoxic T lymphocytes (CTLs), the PD-L1 expression will be induced by IFNγ secreted from CTLs in preparation for an immune attack (Mandai et al., 2016). Therefore, we give exogenous IFNγ to mimic *in vivo* microenvironment. To investigate the involvement of dihydropyridine calcium channel blockers in PD-L1 regulation, we added three LTCC blockers, Lercanidipine, Amlodipine and Azelnidipine, to observe the change of the PD-L1 protein expression stimulated by IFNγ after 24 h of treatment in NCI-H460. To varying degrees, all three compounds inhibited the expression. The relative protein level of PD-L1 was analyzed quantitatively on the right (Figure 1A). In parallel, a similar downregulation of PD-L1 was observed in NCI-H1299 (Figure 1B). This observation was further validated in two cases of primary lung cancer cells (Figure 1C). In order to exclude the influence of calcium channel inhibitors on cell survival, cells were treated with special concentrations of inhibitors (10 μM) for 24 h and cell survival rate was assessed by Sulforhodamine B (SRB) assay. We found that these inhibitors didn’t affect cell growth compared to the control group (Supplementary Figure S1A). Furthermore, we detected that Lercanidipine could down-regulate the PD-L1 protein expression with IFNγ stimulation in a dose-dependent manner in NCI-H1299 (Figure 1D). Considering their efficacy, we prefer to choose Lercanidipine as our tool to continue the next experiments. We then examined the dose-dependent effects of Lercanidipine and further confirmed that Lercanidipine remarkably reduced their expression levels in NCI-H460 (Supplementary Figure S1B). PD-L1 surface expression was the foundation of its biological function. Using flow cytometry, we found that differential concentrations of Lercanidipine reduced the expression levels of cell membrane surface PD-L1 in the proportion cells positive for PD-L1 compared to IFNγ groups after 24 h treatment (Figure 1E). In addition, we also investigated whether the expression of MHC I and CD47, the cell surface immunosuppressive factor, would be influenced by Lercanidipine. The results showed that the cell surface expression of MHC I and CD47 was not significantly affected by Lercanidipine (Supplementary Figure S1C). Collectively, these data suggested that Lercanidipine can inhibit the expression of PD-L1.

### Lercanidipine Inhibits the Transcription of PD-L1

**FIGURE 2:**
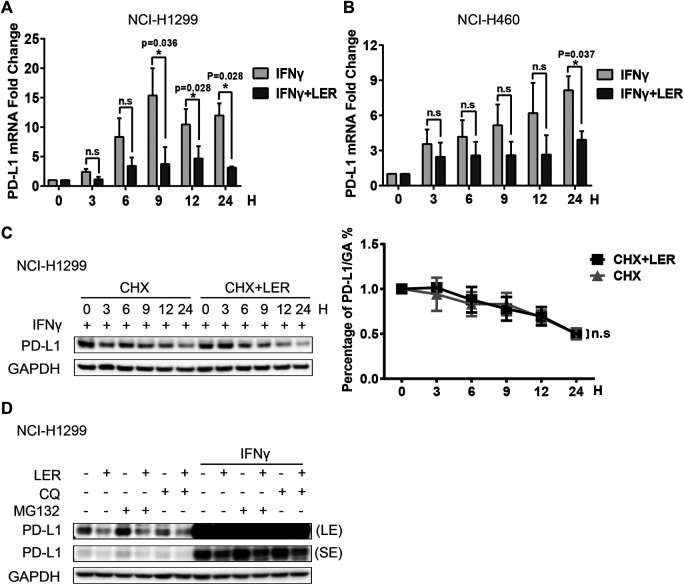
Lercanidipine inhibited the transcription of PD-L1. **(A,B)** NCI-H1299 cells and NCI-H460 cells were treated with IFNγ (10 ng/ml) and Lercanidipine (10 μM) or IFNγ (10 ng/ml) alone for 0, 3, 6, 9, 12, and 24 h, then the relative expression of PD-L1 mRNA was quantified by qRT-PCR. n = 3. **(C)** Western blot of PD-L1 protein in NCI-H1299 cells with or without Lercanidipine in the presence of CHX at 20 μg/mL for 0, 3, 6, 9, 12, and 24 h. The relative protein level of PD-L1 was analyzed quantitatively on the right. n = 3. **(D)** The change of PD-L1 protein was detected in NCI-H1299 treated with Lercanidipine for 24 h with or without IFNγ (10 ng/ml) stimulation meanwhile the cells were treated with CQ (10 μg/mL) or MG132 (10 μM) for 8–10 h. LE, long exposure; SE, short exposure. Data are the mean ± SEM of triplicate experiments. Statistical differences were determined by Student’s t-test. *, *p* < 0.05; n.s: not significant.

Then we explored how Lercanidipine influences the level of PD-L1. Studies have shown that IFNγ could continuously induce PD-L1 expression in 24 h (Moon et al., 2017). Subsequently, we examined the PD-L1 mRNA expression induced by IFNγ, and found that Lercanidipine can reduce the transcriptional levels of PD-L1 in a time-dependent manner in NCI-H1299 cells (Figure 2A). A significant decrease of PD-L1 mRNA expression was observed in NCI-H460 cells (Figure 2B). Next, we examined the stability of PD-L1 proteins. Cycloheximide (CHX) is a compound that inhibits the synthesis of eukaryotic cytoplasmic proteins by impairing ribosomal translocation (Buchanan et al., 2016), which is widely used to determine the half-life of proteins. Therefore, we introduced Cycloheximide to further examine whether Lercanidipine influences the half-life of PD-L1 protein. However, there was no significant difference in the half-time of PD-L1 protein with or without Lercanidipine administration (Figure 2C). To further confirm this effect, cells were treated with Ubiquitin-proteasome pathway inhibitor MG132 and Lysosomal pathway inhibitor Chloroquine (CQ), which can avoid two major ways of protein degradation in the body (Dikic, 2017). We found these inhibitors also can’t reverse Lercanidipine effect on down-regulating PD-L1 proteins (Figure 2D). These data suggested that Lercanidipine inhibits the transcription of PD-L1.

### Lercanidipine Suppresses the Phosphorylation of STAT1

**FIGURE 3:**
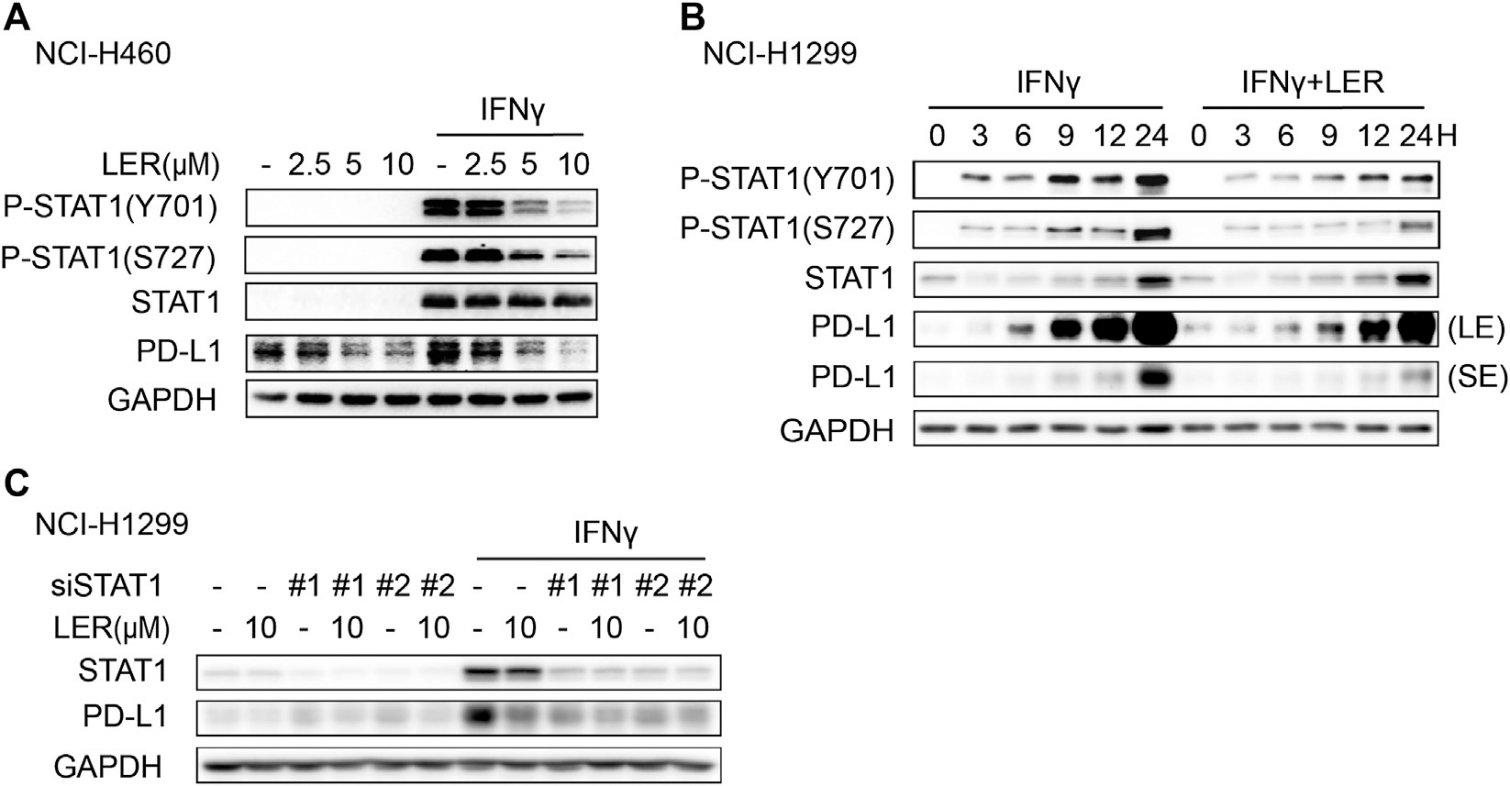
Lercanidipine inhibited the phosphorylation of STAT1. **(A)** NCI-H1299 cells were analyzed by Western blot for P-STAT1, STAT1 and PD-L1 proteins after the treatment of indicated concentrations of Lercanidipine (2.5, 5, and 10 μM) for 24 h. **(B)** The P-STAT1, STAT1 and PD-L1 proteins in NCI-H1299 cells were evaluated with or without Lercanidipine in the presence of IFNγ (10 ng/ml) for 0, 3, 6, 9, 12, and 24 h. LE, long exposure; SE, short exposure. **(C)** The expression of PD-L1 was measured in NCI-H1299 cells transfected with siSTAT1 treated with or without IFNγ (10 ng/ml) and Lercanidipine (10 μM).

Next, we explored how Lercanidipine regulated PD-L1 transcription. The Janus kinase (JAK)-signal transducer and activator of transcription 1 (STAT1) signal axis is activated by IFNγ to induce the expression of genes which display tremendous role in immune system (Ivashkiv, 2018), including PD-L1 (Garcia-Diaz et al., 2017). In addition, the activation of STAT1 requires phosphorylation of tyrosine 701 (Tyr701), which is a key activation step to induce the formation and translocation of STAT1 dimers (Ramana et al., 2002), and serine phosphorylation will further enhance the transcriptional activity (Wen et al., 1995). Consequently, we used Lercanidipine to consider the change of phosphorylation of STAT1. The results indicated that the increase in phosphorylation of STAT1 at Tyr701 and Ser727 induced by IFNγ was inhibited by Lercanidipine (Figure 3A). To further determine this result, we conducted time course studies and found the inhibition of phosphorylation was consistently inhibited in 24 h (Figure 3B). Moreover, we knocked down STAT1 by siRNA and found that the ability of Lercanidipine down-regulating PD-L1 was nearly abolished (Figure 3C). In addition, we also investigated whether the other signaling pathways are involved in the regulation of Lercanidipine on PD-L1 expression. As shown in Supplementary Figure S2A, Lercanidipine significantly down-regulated the level of PD-L1 without affecting the RAS-ERK1/2 and PI3K/mTOR/S6K1 signaling pathways. These data all suggested that Lercanidipine regulates PD-L1 transcription through STAT1.

### Calcium Channel Blockers Down-Regulate the Expression of PD-L1 by Reducing Cytosolic Calcium

**FIGURE 4:**
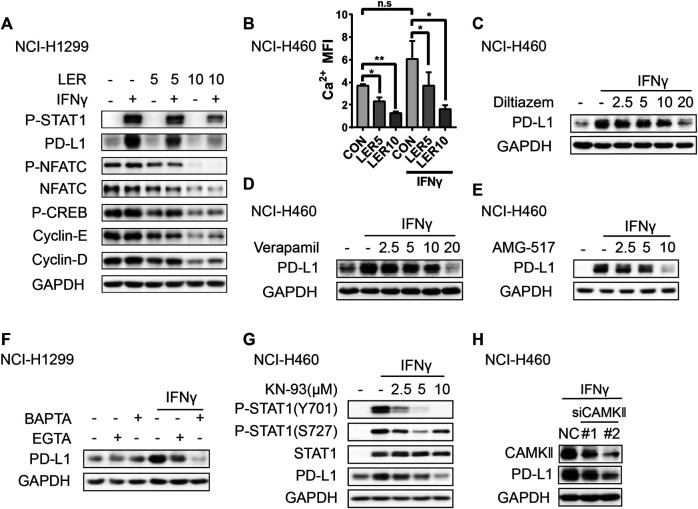
Calcium channel blockers inhibited the expression of PD-L1 through reducing cytosolic calcium. **(A)** NCI-H1299 cells were treated with Lercanidipine (5 and 10 μM) and IFNγ (10 ng/ml) or without IFNγ (10 ng/ml) stimulation for 24 h and protein expression of P-NFATC, NFATC, P-CREB, Cyclin-E, Cyclin-D, P-STAT1 and PD-L1 proteins. **(B)** NCI-H460 cells were treated with Lercanidipine (5 and 10 μM) with IFNγ (10 ng/ml) stimulation or not for 24 h and the calcium MFI was measured by flow cytometry. **(C,D)** The expression of PD-L1 protein was measured by Western blot in NCI-H460 cells when treated with Diltiazem and Verapamil (2.5, 5, 10, and 20 μM) and IFNγ (10 ng/ml) for 24 h. **(E)** NCI-H460 cells induced by IFNγ (10 ng/ml) were treated with AMG-517 (2.5, 5, and 10 μM) for 24 h, and the expression of PD-L1 was detected by Western blot. **(F)** The PD-L1 protein was determined by Western blot after treating with BAPTA (10 μM) and EGTA (100 µM) with or without IFNγ (10 ng/ml) stimulation for 24 h. **(G)** The PD-L1 and P-STAT1 proteins were measured in NCI-H460 treated with KN-93 (2.5, 5, and 10 μM) and IFNγ (10 ng/ml) for 24 h. **(H)** The expression of PD-L1 was measured in NCI-H460 cells transfected with siCAMKⅡ. **, *p* < 0.01; *, *p* < 0.05.

Given that Lercanidipine mainly works through inhibiting Ca^2+^ entry, then we have to validate whether the decrease of cytosolic Ca^2+^ accounted for the inhibition of PD-L1 by Lercanidipine. Generally, the elevation of cytosolic Ca^2+^ promotes the activation of Ca^2+^-dependent signaling enzymes such as calmodulin kinase (CaMK) and calcineurin (CN). Ca^2+^-CaMK is involved in the expression of cyclin D1 by regulating the expression of transcription factors like cAMP-responsive element binding protein (CREB). Calcineurin can dephosphorylate NFATC1 proteins, allowing them enter the nucleus and regulate expression of MYC, cyclin E and E2F (Roderick and Cook, 2008). To confirm this hypothesis, we investigated the change of these signaling and found that these proteins were inhibited by Lercanidipine with or without IFNγ induction, which indicated the inhibition of calcium signaling pathway (Figure 4A). At the same time, we also detected the level of cytosolic calcium and found the similar result (Figure 4B). It’s reported that IFNγ can induce an obvious increase in cytosolic Ca^2+^ (Mizuno et al., 2008; Deng et al., 2018), however, the stimulation of IFNγ didn’t cause the change of calcium signaling related proteins. To further confirm the role of cytosolic calcium, we introduced the other two non-dihydropyridine calcium channel blockers, Diltiazem and Verapamil, to verify the effect on the expression of PD-L1, and they all inhibited the expression of PD-L1 (Figures 4C,D). Like L-type calcium channel, the activation or suppression of other plasma membrane Ca^2+^-permeable ion channels also can induce the fluctuation of calcium, such as Transient Receptor Potential (TRP) channels and Calcium release-activated calcium channel (Monteith et al., 2017). Hence, we used the compound AMG-517, an effective and special TRPV1 antagonist, also showed the same inhibition (Figure 4E). To further examine the role of calcium, we introduced extracellular calcium chelating agent EGTA and intracellular calcium chelator BAPTA-AM. Through verification above two compounds all reduced the expression of PD-L1 to varying degrees, and BAPTA-AM exhibited a better effect (Figure 4F), which suggested cytosolic calcium play a more crucial role. CaMK, the downstream protein kinase of calcium, plays a significant role in the regulation of many cellular processes. CaMKII, activated by calcium, can phosphorylate STAT1 serine residues by interacting directly with STAT1 (Nair et al., 2002). Based on this, we guessed that Lercanidipine may influence the expression of PD-L1 by inhibiting CAMKII signaling. As predicted, we detected that the CaMKII inhibitor KN-93 suppressed the phosphorylation of STAT1 and the expression of PD-L1 (Figure 4G). Furthermore, Knockdown of CAMKII also exhibited the same effect (Figure 4H). Together, these results suggested that Lercanidipine leads to the change of PD-L1 through the reduction of cytosolic calcium.

### Calcium Channel Blockers Enhanced Killing Ability of T Cells

**FIGURE 5:**
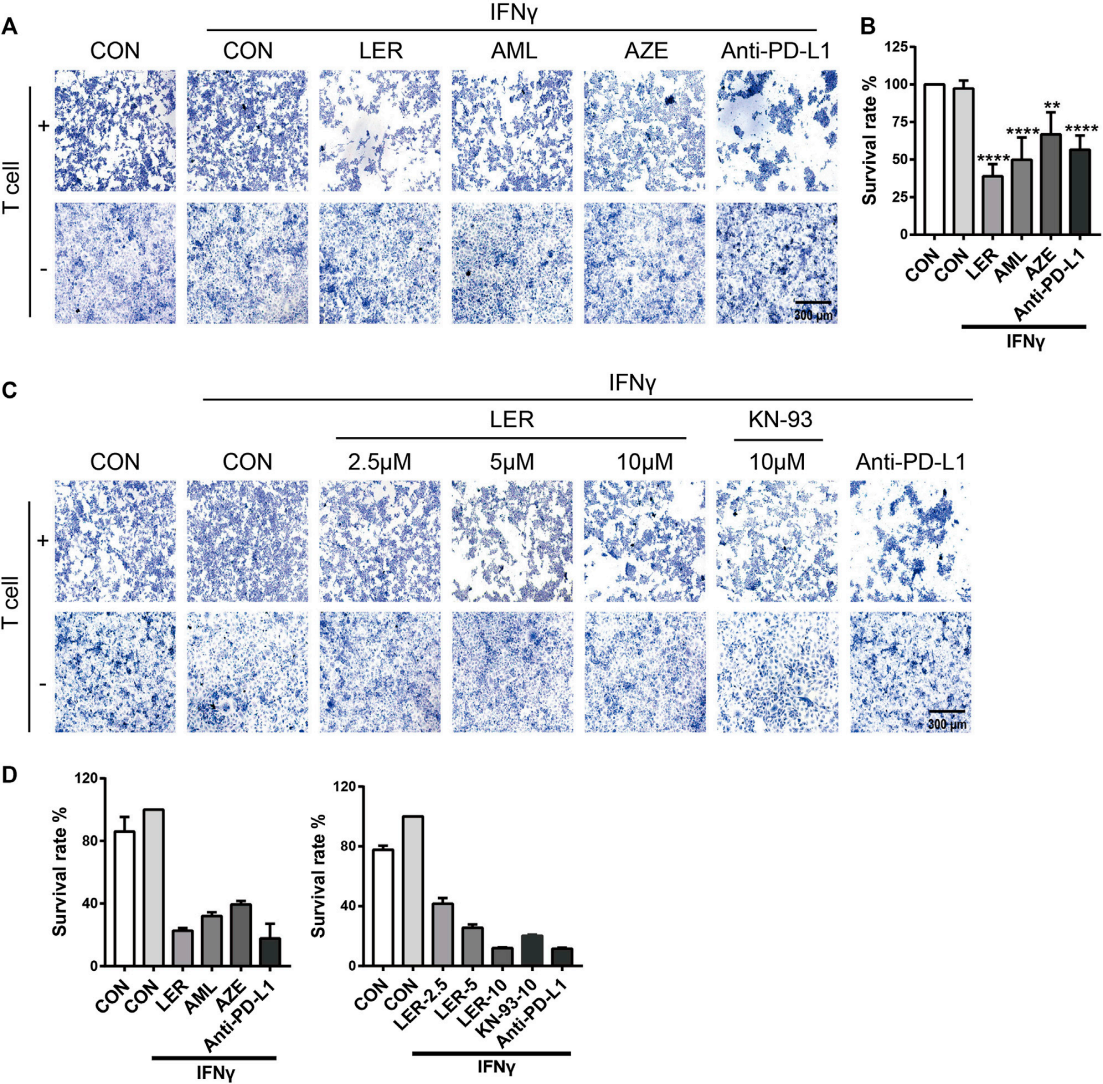
The down-regulation of PD-L1 strengthened the T cell-mediated killing ability. **(A)** T cell killing assay of H1299 cells treated with IFNγ, Lercanidipine, Amlodipine, Azelnidipine and Anti-PD-L1 (positive control) for 24 h. **(B)** T cell killing assay of H1299 cells treated with IFNγ, Lercanidipine, Amlodipine, Azelnidipine and Anti-PD-L1 (positive control) for 24 h. Then H1299 cells survival assay was implemented. **(C)** T cell killing assay of H1299 cells treated with IFNγ, the indicated doses of Lercanidipine (2.5, 5, and 10 μM), KN-93 (10 μM) and Anti-PD-L1 (positive control) for 24 h. **(D)** The images were quantified with Image-Pro Plus 6.0 software. ****, *p* < 0.0001; **, *p* < 0.01.

To determine the biological significance of dihydropyridine calcium channel blockers down-regulating PD-L1 expression, we performed a T cell killing assay using NCI-H1299 cells which were treated with Lercanidipine, Amlodipine and Azelnidipine, at the same time, we set Anti-PD-L1 (50 µg/ml) as positive control. Compared with the negative control, these three calcium channel blockers all induced varying degrees of T cell-mediated cancer cells death, and this result was consistent with efficacy down-regulating PD-L1 (Figure 5A). To further confirm the effects, we conducted the SRB assay to assess the survival of tumor cell in T cell killing assay. A similar result was observed (Figure 5B). Furthermore, Lercanidipine and KN-93 can strengthen the T cell-mediated killing ability in a dose-dependent manner (Figure 5C). The corresponding images were quantified with Image-Pro Plus 6.0 software (Figure 5D). These results all revealed that the decrease of calcium and inhibition of calcium signaling pathways have biological significance.

## Discussion

In the last few years, calcium channel blockers of dihydropyridine have been studied as important antihypertensive drug in clinic. However, we found that they are involved in the regulation of PD-L1. Nowadays, few instances concerning dihydropyridine calcium channel blockers and cancer immune have been reported. Our results show that dihydropyridine calcium channel blockers suppress the transcription of PD-L1 by inhibiting calcium signaling, which indicate the potential connection of calcium signaling and immune regulation.

Calcium, one of the most significant second messengers, plays a crucial role in gene transcription. The elevation of calcium will activate extensive downstream calcium signaling pathway including Calcineurin which de-phosphorylates NFATC1 proteins and promotes NFAT to regulate relative gene transcription (Crabtree, 2001; Hogan et al., 2003). Moreover, calcium also can encourage CaMKII to the cell nucleus to drive CREB to phosphorylation and gene expression (Ma et al., 2014). CREB-binding Protein and p300/CBP-associated Factor can activate the transcription of P53 protein (Scolnick et al., 1997). Although the C terminus of the L-type voltage-gated calcium channel can mediate the expression of genes, most genes relate to neuronal signaling (Gomez-Ospina et al., 2006). Moreover, our results (Figures 4A,C–F) found several kind of calcium channel blockers and chelators all play a similar role in the regulation of PD-L1, so we are inclined to the role of calcium. Notably, we found that calcium signaling involves in the transcription of PD-L1 in cancer cells, which links calcium signaling with immune regulation.

The transcription of PD-L1 is regulated by many signaling pathways which have been studied widely (Atsaves et al., 2017; Zerdes et al., 2018; Zhu et al., 2018). IFNγ, one of cytokines, activates STAT1 and STAT3 with comparable efficiency, and induced STAT1 response is more strong than STAT3 response (Qing and Stark, 2004). Thus, we are inclined to investigate the change of STAT1 with Lercanidipine. Reportedly, IFNγ can cause increasement of cytosolic Ca^2+^, however, our result (Figure 4A) didn’t exhibit this trend, maybe the cells we used was different or the concentration we used didn’t arrive at the point that elicits a rapid increase of cytosolic Ca^2+^.

CaMK, one kind of serine-threonine kinases, can phosphorylate serine 727 (Ser727) of STAT1 by interacting directly with STAT1 in NIH 3T3 (Nair et al., 2002). Theoretically, the phosphorylation of STAT1 at tyrosine 701 (Tyr701) cannot be phosphorylated by CaMK, at least cannot be directly accomplished. Furthermore, our results (Figure 4G) showed that the CaMK inhibitor KN-93 strongly suppressed the phosphorylation of STAT1 at Tyr701 with IFNγ stimulation in a concentration-dependent manner, which indicated that CaMK is actually involved in the phosphorylation of tyrosine 701. Pyk2, a tyrosine kinase, can be regulated by calcium and CaMK (Wang et al., 2008), and the interaction of Pyk2 and JAK kinases has been confirmed (Lev et al., 1995; Miyazaki et al., 1998). The published reports also pointed that CaMK can accomplish tyrosine phosphorylation by mediating relative tyrosine kinases like Pyk2, however, whether Pyk2 or other tyrosine kinases take part in the regulation of tyrosine phosphorylation and how CaMK affects these signaling pathways in cancers also need further investigation. Here, we proposed that CaMK induces directly or indirectly tyrosine and serine phosphorylation of STAT1, and its’ meaning to the regulation of immune checkpoints worth further exploration.

In general, our study indicates that Lercanidipine can modulate the transcription of PD-L1 by inhibiting calcium related signaling pathways. Our findings not only provide a thinking about reducing cytosolic Ca^2+^ thereby inhibiting PD-L1 in cancer cells, but also provide novel ideas to improve clinical immunotherapy.

## Data Availability Statement

All datasets generated for this study are included in the article/Supplementary Material.

## Author Contributions

LD designed the study. XC revised the article. XP and RL carried out the experimental studies and wrote the manuscript. HG and WZ conducted the data analysis. HG, WZ, and XX revised the manuscript.

## Funding

This work was supported by the National Natural Science Foundation of China (No. 81930102 to B. Yang) and the National Natural Science Foundation of China (No. 81773754 to LD).

## Conflict of Interest

The authors declare that the research was conducted in the absence of any commercial or financial relationships that could be construed as a potential conflict of interest.
