# Supplementary material for: Dihydropyridine Calcium Channel Blockers Suppress the Transcription of PD-L1 by Inhibiting the Activation of STAT1
Source: Front Pharmacol. 2021 Jan 13;11:539261. doi: 10.3389/fphar.2020.539261 (PMC7838064; doi:10.3389/fphar.2020.539261)
Supplement: Supplementary file 1 [file datasheet1.docx]

**Supplementary Figures**

The following are Supplementary data to this article:


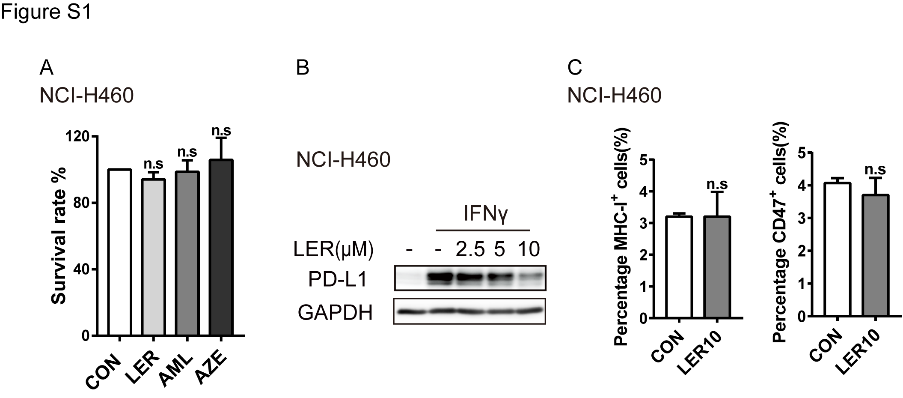


**Supplementary Figure.1 A** NCI-H460 cell survival assay was implemented in special concentration of inhibitors (10μM) for 24 hours. n=4. **B** NCI-H460 cells were treated with Lercanidipine (2.5μM, 5μM and 10μM) for 24 hours，and the expression of PD-L1 was analyzed by Western blot. **C** Surface MHC-Ⅰ and CD47 expression on NCI-H460 treated with Lercanidipine (10μM) was determined by flow cytometry. Cells were estimated for MHC-Ⅰ, CD47 or mouse IgG control antibody. Data were the mean ± SEM of triplicate experiments. The data were analyzed by one-way ANOVA with Dunnett’s post hoc test. *n.s*: not significant.

**
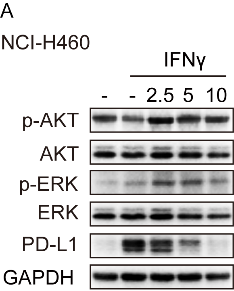
**

**Supplementary Figure.2 A** NCI-H460 cells were treated with Lercanidipine (2.5μM 5μM and 10μM) and IFNγ (10ng/ml) stimulation for 24 hours and protein expression of p-AKT, AKT, p-ERK, ERK and PD-L1 proteins.

**Supplementary Tables:**

| **Gene name** | | **Primer Sequence(5’—3’)** |
| --- | --- | --- |
| Negative control siRNA | | UUCUCCGAACGUGUCACGUTT |
| *STAT1* | siRNA#1 | CCCUAGAAGACUUACAAGAUGAAUA |
|  | siRNA#2 | GCGGAGACAGCAGAGCGCCUGUAUU |
| *CAMKⅡ* | siRNA#1 | UCAGAUUCUGGAGAGUGUUAA |
|  | siRNA#2 | UUGAGGCCUACACGAAGAUUU |

Table S1. siRNA sequences

| **Gene name** | | **Sequence** | |
| --- | --- | --- | --- |
| *CD274* | #1 | Forward | 5’-TCACTACACAGCCCTCCTAA-3’ |
|  |  | Reverse | 5’-ACACCAGAATATGGCCAAGAG-3’ |
|  | #2 | Forward | 5’-CCCTAATTTGAGGGTCAGTTCCT-3’ |
|  |  | Reverse | 5’-CTCAGTCATGCAGAAAACAAATTGA-3’ |
|  | #3 | Forward | 5’-CTGAACGCATTTACTGTCACGG-3’ |
|  |  | Reverse | 5’-GGTCTTCCTCTCCATGCACA-3’ |
| *ACTIN* | | Forward | 5’-ATTCCTATGTGGGCGACGAG-3’ |
|  |  | Reverse | 5’-CCAGATTTTCTCCATGTCGTCC-3’ |

Table S2. Human primers used in quantitative real time-PCR
